# Predictive Machine Learning Model to Assess the Adsorption Efficiency of Biochar-Heavy Metals for Effective Remediation of Soil–Plant Environment

**DOI:** 10.3390/toxics12080575

**Published:** 2024-08-07

**Authors:** Xiang Li, Bing Chen, Weisheng Chen, Yilong Yin, Lianxi Huang, Lan Wei, Mahrous Awad, Zhongzhen Liu

**Affiliations:** 1 Key Laboratory of Plant Nutrition and Fertilizer in South Region, Ministry of Agriculture, Guangdong Key Laboratory of Nutrient Cycling and Farmland Conservation, Institute of Agricultural Resources and Environment, Guangdong Academy of Agricultural Sciences, Guangzhou 510640, China; 13538631591@163.com (W.C.); yilong516@163.com (Y.Y.); hlx4@163.com (L.H.); 13450364816@163.com (L.W.); 2 Institute of Animal Science, Guangdong Academy of Agricultural Sciences, Collaborative Innovation Center of Aquatic Sciences, Key Laboratory of Animal Nutrition and Feed Science in South China, Ministry of Agriculture and Rural Affairs, Guangdong Provincial Key Laboratory of Animal Breeding and Nutrition, Guangzhou 510640, China; chenbing114@163.com; 3 Department of Soils and Water, Faculty of Agriculture, Al-Azhar University, Assiut 71524, Egypt; mahrousawad.4419@azhar.edu.eg

**Keywords:** biochar, heavy metal, transformation, adsorption, predictive models

## Abstract

Biochar is crucial for agricultural output and plays a significant role in effectively eliminating heavy metals (HMs) from the soil, which is essential for maintaining a soil–plant environment. This work aimed to assess machine learning models to analyze the impact of soil parameters on the transformation of HMs in biochar–soil–plant environments, considering the intricate non-linear relationships involved. A total of 211 datasets from pot or field experiments were evaluated. Fourteen factors were taken into account to assess the efficiency and bioavailability of HM–biochar amendment immobilization. Four predictive models, namely linear regression (LR), partial least squares (PLS), support vector regression (SVR), and random forest (RF), were compared to predict the immobilization efficiency of biochar-HM. The findings revealed that the RF model was created using 5-fold cross-validation, which exhibited a more reliable prediction performance. The results indicated that soil features accounted for 79.7% of the absorption of HM by crops, followed by biochar properties at 17.1% and crop properties at 3.2%. The main elements that influenced the result have been determined as the characteristics of the soil (including the presence of different HM species and the amount of clay) and the quantity and attributes of the biochar (such as the temperature at which it was produced by pyrolysis). Furthermore, the RF model was further developed to predict bioaccumulation factors (BAF) and variations in crop uptake (CCU). The R^2^ values were found to be 0.7338 and 0.6997, respectively. Thus, machine learning (ML) models could be useful in understanding the behavior of HMs in soil–plant ecosystems by employing biochar additions.

## 1. Introduction

Heavy metal (HM) contamination has become an emerging environmental issue worldwide [1]. Excessive HMs in soils may pose risks to humans through their presence in the food chain, which has attracted academic attention and public concerns [2]. A range of adverse health effects has been reported, such as lung cancer, bone fractures, and kidney dysfunction [3]. The most effective method for decreasing crop uptake of HMs is the use of in situ remediation techniques to reduce the bioavailability of HMs in soil–plant environments [4]. Numerous materials have been evaluated for the immobilization of HMs [5,6,7]. In recent years, biochar has been regarded as a promising material that can reduce the bioavailability and mobility of HMs in contaminated soils [8]. Biochar is a carbon-rich product that is heated in a closed container under limited air. It has the advantages of low cost and enhancement of soil nutrients and crop production [9] and has a great potential to reduce the transformation of HMs in soils [10,11]. 

Biochar efficiency is significantly dependent on multiple variables, such as raw materials, pyrolysis conditions, and residence time, which cause different biochar characteristics. Guo et al. [12] reported that pH and soil organic carbon factors affect the bioavailability of HMs in soils. Biochar could change soil properties such as pH and organic carbon and significantly change the soil extractable HMs [13]. In the use of biochar for the remediation of HM-contaminated soils, it is critical to consider the key factors determining the effectiveness of the biochar to adjust the right direction for HM reduction in the soil–plant environment. Biochar has a strong sorptive or interactive capacity due to its highly functionalized surface [14]. Its attractive properties make it a promising material for a multitude of applications, including species assimilation and spontaneous preliminary concentration [15]. The process by which biochar exerts its influence on the qualities of soil is an essential component in determining the extent of its advantages and the compromises associated with its use [16]. As a method for cleaning up polluted soil, biochar has recently garnered a significant amount of attention [17]. The farming industries have been able to expedite the development of biochar implementation on a large scale as an effective tool for crop production and decontamination [18]. In general, the conventional experimental method is control-variable; it is not in favor of identifying the relationships between biochar and HMs transformation. Therefore, the use of biochar modeling in a strategic approach and the execution of the model’s predictions.

Studies have shown that biochar may enhance soil properties and influence soil redox reactions. Biochar has the capacity to significantly reduce the movement and accessibility of HMs in polluted soils, hence decreasing their absorption by crops and ultimately enhancing plant development [16]. The rhizosphere soil undergoes modification due to the influence of root exudates, resulting in an attainable zone, whereas the movement of HMs may be changed. Root exudates excrete H+ ions and organic acids, acidifying the rhizosphere and increasing plant nutrient absorption [17]. The addition of biochar to polluted soil has been proposed as a potential technique for immobilizing HMs. Biochar has a higher cation exchange capacity, more functional groups, more microporosity, and a larger surface area, all of which contribute to its improved performance [14]. The diversity and characteristics of biochar and complex soils affect the HM adsorption ratio in the soil. Soil HM immobilization efficacy varies greatly depending on the cation exchange capability and organic matter content. Due to the significant interactions between soil pH and biochar oxygen concentration, biochar surface functional molecules may alter the HM adsorption fraction in soils [15]. Conducting scientific experiments to study the limits of HM immobilization ratios is a tedious, laborious, and costly approach. It is necessary to devise a novel approach in order to have a deeper comprehension of these aspects.

The machine learning (ML) method is an emerging tool to elaborate complicated multivariate relationships, especially with the rapid development of interpretable ML models and interpretation methods [19,20,21]. ML is able to learn the relationships between the input variables and output variables from a training dataset; the relationships can used to predict the new case. The application of the ML technique has been successfully demonstrated to have the ability to predict the pollutant’s mobility in soils [22]. ML methods have been used to predict HMs immobilization in biochar-amended soils [23]. However, machine learning is limited by the complexity of the relationship between biochar and soil properties. Different input sets had a significant impact on the results of the feature analysis. There is a lack of general models to predict the relative contribution of biochar amendment in the soil–plant environment. Thus, a data-driven methodology is needed to explore the effectiveness of biochar based on different input variable sets. 

The present research investigation employed machine learning methods for predicting the efficacy of biochar-HMs in immobilizing within the soil–plant environment. In this study, four methods, namely linear regression (LR), partial least squares (PLS), support vector regression (SVR), and random forest (RF), were applied to build a predicting model for evaluating the effectiveness of biochar in a soil–plant environment. Overall, 211 sets of experimental data from plant or field investigations were assessed. To forecast the bioavailability and immobilization efficiency of biochar amendments for HMs, 14 influencing factors were identified. This study aimed to perform the following: (1) evaluate the ML models for predicting HM immobilization efficiency in biochar-amended soils; (2) compare the predicting accuracy of ML models with traditional models; and (3) explore the influence of input variables sets on prediction accuracy and feature analysis. 

## 2. Material and Methods

### 2.1. Data Collection and Modeling Methods

The experimental data of the biochar application for HM remediation in soil–plant environments were collected from the previous literature [24,25,26,27,28,29,30,31,32,33,34,35,36,37,38,39,40,41,42]. The data were extracted directly from the tables in the published literature to form graphs using the Web Plot Digitizer Software (https://apps.automeris.io/wpd/, accessed on 4 July 2024). More details are given in Supplementary Materials. A total of 211 sets of experimental data from pot or field experiments were evaluated. To predict immobilization efficiency and bioavailability of biochar amendments for HMs, the following 14 influencing factors were considered (X): Pot experiment or filed experiment (X1); type of crops (X2); application rate (X3); feedstock (X4); pyrolysis temperature (X5); clay content (X6); silt content (X7); sand content (X8); soil pH (X9); soil organic carbon (X10); crop duration (X11); total HMs in soil (X12); available HMs in soil (X13); and species of HM in soils (X14). Furthermore, influenced factors were divided into three segments as follows: (a) soil characteristics (X6, X7, X8, X9, X10, X12, X13, and X14); (b) biochar characteristics (X3, X4, and X5); and (c) crop characteristics (X1, X2, and X11). 

The Python language was used to call linear regression (LR), partial least squares (PLS), support vector regression (SVR), and random forest (RF) from the scikit-learn (https://scikit-learn.org/stable/ (accessed on 4 January 2022)) for modeling. The detailed methods are given in the supplementary data section. 

### 2.2. Data Analyzing

The immobilization efficiency was used to investigate the impact of biochar on mobility of HMs and calculated using the following equation [43]:

(1) $$Immobilization\;efficency\;(IE)=\frac{C_{soil}-C_{biochar+soil}}{C_{soil}}\times 100\%$$

where $C_{soil}$ is the available concentration of HMs in soils before the biochar amendments, and $C_{biochar+soil}$ is the available concentration of HMs in soils after biochar amendments. 

The change in crop uptake and bioaccumulation factors were used to evaluate the impact of biochar on HMs bioavailability in the soil–crop systems and calculated by the following equations [25]:

(2) $$Change\;in\;crop\;uptake\;(CCU)=\frac{\;C_{crop}-C_{biochar+crop}}{C_{crop}}\times 100\%$$

where $C_{crop}$ is the concentration of HMs in crops before the biochar amendment, and $C_{biochar+crop}$ is the concentration of HMs in crops after biochar amendments.

(3) $$Bioaccumulation\;factors\left(BAFs\right)=\frac{C_{crop}^{\prime }}{C_{soil}^{\prime }}\times 100\%$$

where $C_{soil}^{\prime }\;$ is the concentration of HMs in soils, $C_{crop}^{\prime }$ is the concentration of HMs in crops.

### 2.3. Linear Regression (LR) 

Linear regression is a regression analysis that uses the least square function, and called the linear regression equation, to model the relationship between one or more independent and dependent variables [44]. This function is a linear combination of one or more model parameters called regression coefficients. The condition with only one independent variable is called simple regression, and the condition with more than one independent variable is called multiple regression.

### 2.4. Partial Least Squares (PLS) 

The partial least squares method is a multivariate statistical data analysis method. The PLS method projects the high-dimensional data space of the independent variable and dependent variable into the corresponding low-dimensional space to obtain the mutually orthogonal eigenvectors of the independent variable and dependent variable, respectively, and then establishes the univariate linear regression relationship between the eigenvectors of the independent variable and dependent variable [45]. Partial least squares regression analysis focuses on the characteristics of principal component analysis, canonical correlation analysis, and linear regression analysis.

### 2.5. Support Vector Regression (SVR) 

SVR is an important application branch of SVM (support vector machine), and SVM itself is proposed for binary classification [46]. SVR is a regression method where the optimal hyperplane does not seek the “most open” of two or more types of sample points like SVM; it seeks the minimum total deviation of all sample points from the hyperplane instead. Support vector regression uses the idea of support vector and Lagrange multiplier to regression analyze the data when fitting. Compared with the least square method, SVR has no limitation in that it can only be used for linear regression, and it is also suitable for nonlinear models. At the same time, the least squares method has a poor regression effect for variables with multicollinearity; support vector regression does not need to worry about multicollinearity.

### 2.6. Random Forest (RF) 

Random forest is an algorithm that uses multiple decision trees to train, classify, and predict samples. It is mainly used in regression and classification scenarios [47]. While classifying the data, RF can also give the importance score of each variable and evaluate the role of each variable in classification. Random forest is a well-known ensemble learning method, which belongs to the part of the ensemble learning algorithm where there is no dependency between weak learners. Because of this advantage, it can run in parallel. Random Forest Regressor is almost the same as RF classifier. During prediction, the results of all trees are averaged to obtain the final prediction result value. RF training can be parallelized and fast. After training, the contribution of each feature to the output can be given. At the same time, it has scale invariance and does not need feature scaling. RF is not as easy to overfit as a decision tree, as RF is insensitive to missing values and is very robust.

### 2.7. Models’ Predictability and Generalizability

The original data was randomly divided into 10 equal sub-samples. Approximately 7.5 sub-samples (training data) were used to optimize the model using LR, PLS, SVR, and RF algorithms. However, the remaining 2.5 sub-samples (testing data) were used to test the evaluation of the model. Additionally, the GridSearchCV method was used for five-fold cross-validation to select for the optimal results and parameters for the study. Model accuracy was evaluated using the regression coefficient (R^2^), mean squared error (MSE), root mean squared error (RMSE), and mean absolute error (MAE). The following are the mathematical representations.

(4) $$R^{2}=1-\frac{\sum_{i=1}^{N}{\left(Y_{i}^{exp}-Y_{i}^{pred}\right)}^{2}}{\sum_{i=1}^{N}{\left(Y_{i}^{exp}-Y_{ave}^{exp}\right)}^{2}}$$

(5) $$MSE=\frac{1}{N}\sum_{i=1}^{N}{\left(Y_{i}^{exp}-Y_{i}^{pred}\right)}^{2}\;$$

(6) $$RMSE=\sqrt{\frac{1}{N}}\sum_{i=1}^{N}({Y_{i}^{exp}-Y_{i}^{pred})}^{2}$$

(7) $$MAE=\frac{1}{N}\sum_{i=1}^{N}{\left(Y_{i}^{exp}-Y_{i}^{pred}\right)}^{2}$$

## 3. Results and Discussion

### 3.1. Comparison of Biochar, Soil, and Crops in the Pearson Correlation Matrix 

**Figure 1:**
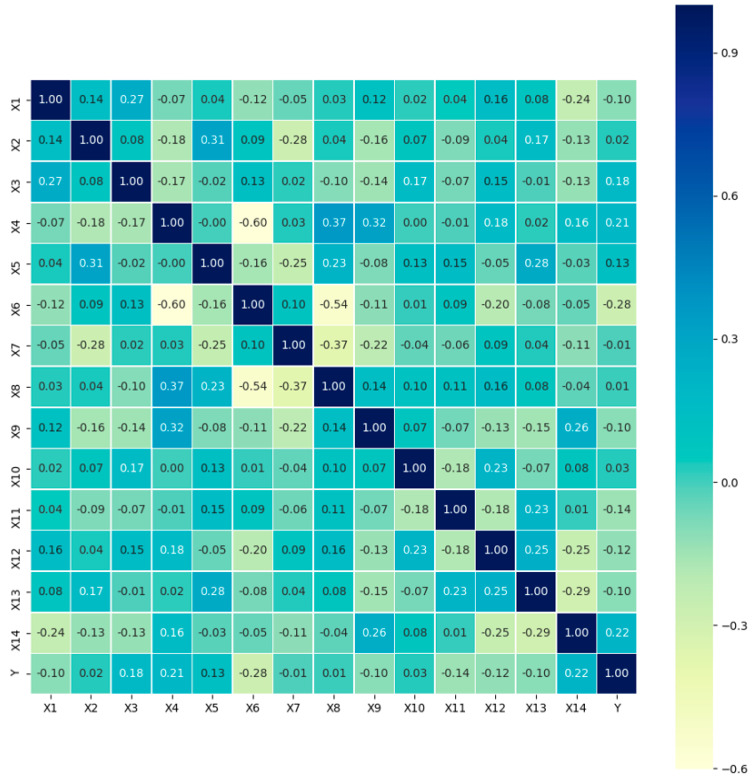
Pearson’s correlation matrix of influencing factors. (X1: experiment scale, X2: type of crops, X3: application rate (%), X4: feedstock, X5: pyrolysis temperature, X6: clay content, X7: silt content, X8: sand content, X9: soil pH, X10: soil organic carbon, X11: crop duration, X12: total heavy metals in soil, X13: available heavy metals in soil, X14: type of heavy metals, and Y: immobilization efficiency).

Pearson’s correlation matrix is given in the supplementary data (Tables S1–S3) for biochar, crops, and soil characteristics. Results indicated that the applied biochar content ranged between 0.22 and 10%, with a slight amount reaching 5%. It has been reported that a higher rate of biochar application probably has no impact on the immobilization of HMs [48]. Furthermore, biochar is the most effective remediation agent for HMs when applied at a rate of below 5%. According to the results, the pyrolysis temperature ranged between 300 and 750 °C, although 500 °C was found to be more significant due to obtaining significant results. HMs were found to be highly susceptible to adsorption on the biochar that was generated at high temperatures due to its large specific surface and reduction in functional groups [49]. Furthermore, the results indicated that 92% of biochar was generated from lignocellulosic biomass, while 8% was derived from animal waste. According to the results of the assessment of HM content in soils, the average content of the HMs in soils followed the following order: Pb (996.26 mg/kg) > Zn (684.96 mg/kg) > Cu (347.83 mg/kg) > Cd (24.05 mg/kg). It was found that most of the HMs exceeded the national threshold levels [50]. It was found that pH values were observed in the soils, ranging from 4.71 to 8.06, while 80% were determined to be lower than 7. It has been suggested in several studies that biochar is usually alkaline and, as a result, could increase soil pH [51]. Adding biochar to acidic soils can raise the pH of the soil, which results in HM precipitates forming, more effectively immobilizing HM [52]. According to the results of crop characteristic experiments, pot experiments accounted for 94% of the results, while field experiments contributed 6%. Crop species were found with the following sequence: vegetable (66%) > corn (19%) > wheat (11%) > rice (4%). Additionally, it has been found that the average crop duration was less than 90 days for the entire crop season. Pearson’s correlation matrix illustrating all the influence factors before modeling is shown in Figure 1. It was found that there was a non-significant correlation observed between the two variables, and the greatest absolute value was less than 0.60. The results showed that all the input parameters were independent variables in the model. 

### 3.2. Statistical Analysis of the Prediction Models 

HM immobilization efficiency was evaluated using four different models (LR, PLS, SVR, and RF) to determine the effectiveness of biochar in improving HM immobilization.

Linear regression is a statistical technique that utilizes the least squares function, known as the linear regression equation, to establish a mathematical model representing the connection between independent and dependent variables. Regression coefficients are model parameters that are combined linearly. Partial least squares regression analysis is a technique used in multivariate statistical data analysis. It involves transforming the high-dimensional data space of the independent and dependent variables into a lower-dimensional space. This transformation allows us to obtain mutually orthogonal eigenvectors for both the independent and dependent variables. The text focuses on the attributes of principal component analysis, canonical correlation analysis, and linear regression analysis. SVR is a significant subdivision of SVM that is specifically designed for binary classification tasks. The analysis of data during fitting is conducted using the concepts of support vector and Lagrange multiplier. SVR, unlike the least square approach, does not have any limits and is well-suited for nonlinear models. RF is a machine learning technique that uses a collection of decision trees to train, categorize, and make predictions on data. The method is fast, parallelized, and scale-invariant. It is not affected by missing values and is persistent.

The statistical parameters of the models used to predict the immobilization efficiency of HMs by biochar amendment are presented in Table 1, including mean error (MSE), average root mean square error (RMSE), mean absolute error (MAE), and coefficient of determination (R^2^). Results indicated that the R^2^ of the RF model for immobilization efficiency in soils was 0.5924, which was significantly higher than that of the SVR model (R^2^ = 0.2421), LR model (R^2^ = 0.2785), and PLS model (R^2^ = 0.3007). It shows that the RF model has a statistically significant advantage over the LR, the PLS, and the SVR models in terms of statistical parameter evaluations. According to Table 1, MSE, RMSE, and MAE for the RF were 0.0596, 0.2441, and 0.1556, respectively, which were the lowest values among the parameters. According to the results, RF models are superior due to their ability to deal with complex non-linearities. Hu et al. assessed the RF model, gradient-boosted machine model (GBM), and generalized linear model (GLM) to predict HM transfer in the soil–crop system and found that the RF model produced the most accurate prediction of BAFs [53]. The higher prediction accuracy is due to the fact that the RF model has been developed directly from the available experimental data in the literature without relying on the underlying assumptions of the traditional model. Bootstrapping the original training data was used to form a decision tree by randomly selecting parts of the features. 

**Figure 2:**
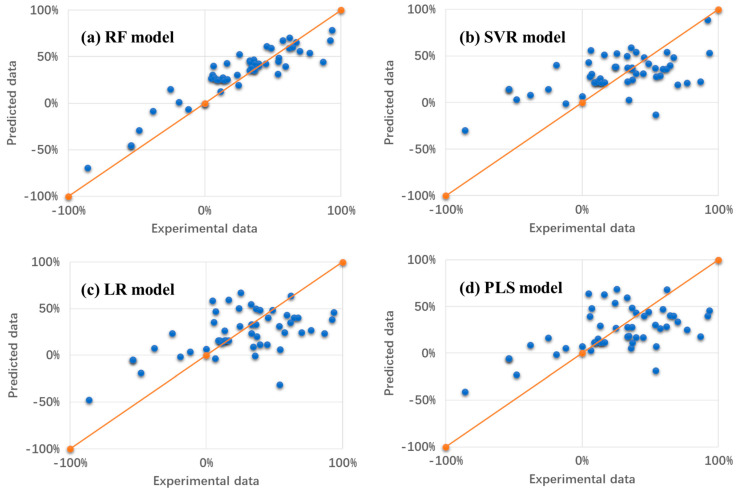
Analysis of the experimental and predicted data based on the model. (**a**) random forest (RF) model, (**b**) support vector regression (SVR) model, (**c**) linear regression (LR) model and (**d**) partial least squares (PLS) model. The red lines refer to the line y = x (45° line).

The cross plots of model predictions versus experimental values are presented in Figure 2 for visual comparison. A comparison of the four models indicates that the proposed model has a superior and more reliable prediction ability, as evidenced by the tighter cloud of points surrounding the 45° line in Figure 2a. The results indicated that RF models were more effective for predicting the impact of biochar amendments on the immobilization efficiency of HMs in soil–crop systems. It is observed from Figure S1 that the deviations between the RF model predictions and the corresponding experimental values are calculated based on the RF model predictions. The results showed that the RF model had a higher performance for immobilization efficiency within the range of 5–70%. Several factors may have contributed to these findings, including the limited datasets available regarding lower and higher immobilization efficiency in the previous literature.

### 3.3. Potential Controls of Soil, Biochar, and Corp Characteristics for HMs Immobilization

**Figure 3:**
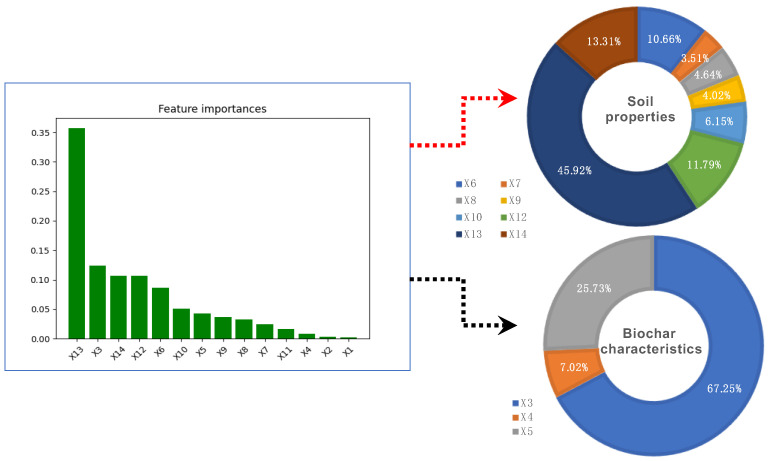
Relative importance of each variable on the immobilization efficiency (X1 is experiment size; X2 is type of crops; X3 is application rate (%); X4 is feedstock; X5 is pyrolysis temperature; X6 is clay content; X7 is silt content; X8 is sand content; X9 is soil pH; X10 is soil organic carbon; X11 is days from planting to sowing; X12 is total heavy metals in soil; X13 initial heavy metals in soils; X14 species of heavy metals).

The results revealed that the RF model was the most accurate in predicting the HM immobilization efficiency of biochar, and the relative significance of each variable was also identified. The mean decrease impurity method was applied to calculate the relative significance of soil, biochar, and crop characteristics on the HM immobilization efficiency. The contribution of each influence variable factor in the model is shown in Figure 3. 

Soil characteristics were found to significantly influence immobilization efficiency, accounting for 79.7% of the total. Previous studies have found that different soil properties, such as pH, soil organic matter, and clays, influence the mobility and bioavailability of HMs in soil–crop systems [54]. Results have suggested that the soil properties were contributed mainly by the available HMs in the soil. Biochar is generally effective for the immobilization of HMs from soils due to direct mechanisms of interaction between HMs and biochar, such as adsorption, precipitation, and complexation. The available HM concentration in soil influences these interactions significantly [53]. Furthermore, it was also found that the species of HM in soils contributed to the effectiveness of the immobilization of biochar [55,56,57]. The biochar exhibited a more vital ability to complex Cu(II) and Pb(II) than Cd(II), resulting in more specific adsorption of Cu(II) and Pb(II) by amended soils than Cd(II) [58]. In this study, the species of HMs contributed about 13.3% to soil properties. Additionally, it was noted that soil clay (10.66%) exhibited a greater effect on immobilization efficacy than soil organic content (6.15%). It has been suggested that clay can increase the micropore area in biochar, resulting in a greater ability to eliminate HMs [59]. Similarly, Ramola et al. [60] reported significant Pb removal from water using a biochar-clay composite in comparison with the use of BC alone. Meanwhile, Jing Y et al. [61] demonstrated a 35% improvement in biochar–clay composites compared with BC alone for the removal of Cd. 

A comprehensive analysis of the 211 published data sets on the immobilization process revealed that biochar characteristics accounted for 17.1% of the immobilization process’ efficiency. It was determined that 67.25% of the contribution could be attributed to the rate at which biochar was applied, suggesting that the application rate of biochar had a positive correlation with biochar amendment. Previous studies have found that the higher the biochar dosage, the greater the increase in soil organic carbon [44]. HMs have changed solid-solution partitioning with the increasing biochar dosage; therefore, their leaching potential in soils has changed. Zhang et al. [62] compared the effects of two levels of biochar on soil Cd reduction and found that the immobilization efficiency for 15 t ha^−1^ of biochar was 34.5%. Studies have also shown that the efficiency of immobilization has not changed significantly as the dosage of biochar has increased to 30 t ha^−1^. In this context, it appears that the dosage of biochar and immobilization efficiency are not linearly related. Based on the results of the RF model, it was determined that biochar dosage was an influential factor in the biochar amendment process. Biochar amendment was also influenced by the pyrolysis temperature, as shown in Figure 3. It contributed to approximately 25.73% of the characteristics of biochar. Rafique et al. [63] reported that the biochar pyrolyzed at 700 °C had greater efficacy in immobilizing HMs than pyrolyzed at 300 °C. The results of previous studies have indicated that increasing the pyrolysis temperature increased specific surface area, porosity, pH, carbon, and ash content, as well as the immobilization efficiency of HMs [64]. However, changing the CEC and volatile matter content altered the immobilization efficiency [65]. Additionally, crop characteristics only accounted for 3.1% of the efficiency of immobilization. It was found that the type of crops and the days from planting to harvesting did not affect HM immobilization efficiency.

### 3.4. RF Model Development for Predicting HMs Bioavailability with Biochar Application

**Scheme 1:**
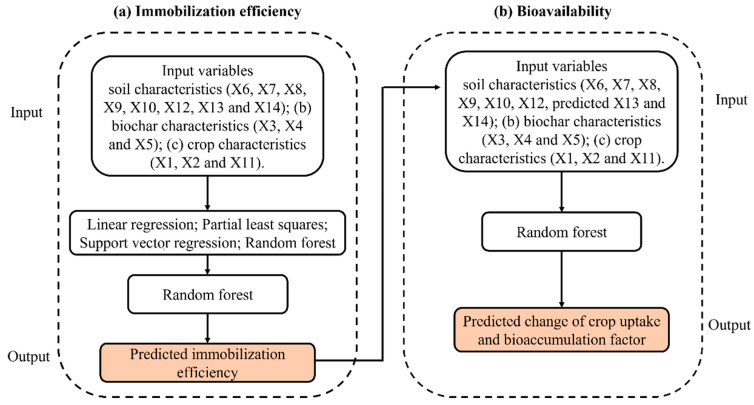
A two-stage approach to predict bioavailability (**a**) random forest model was used to predict the immobilization efficiency. (**b**) The predicted available HM concentrations are used as inputs for a developed random forest model to predict changes in crop uptake and bioaccumulation factors. (X1 is experiment size; X2 is type of crops; X3 is application rate (%); X4 is feedstock; X5 is pyrolysis temperature; X6 is clay content; X7 is silt content; X8 is sand content; X9 is soil pH; X10 is soil organic carbon; X11 is days from planting to sowing; X12 is total heavy metals in soil; X13 initial heavy metals in soils; X14 type of heavy metals).

**Figure 4:**
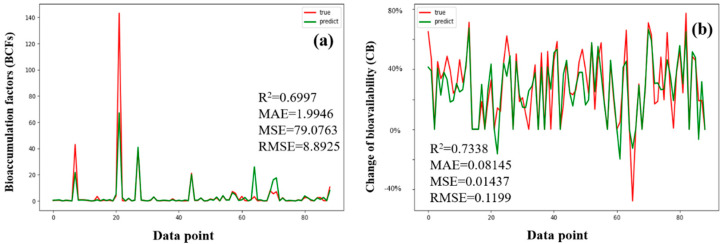
RF model for experimental and predicted values versus the number of data points: (**a**) bioaccumulation factors (BAFs) and (**b**) change in crop uptake (CCU); the red line represented true data, and the green line represented the predicted data.

Biochar has been found to be an effective way to reduce plant uptake of pollutants due to its ability to decrease HMs in the soil and reduce HM uptake by crops [4]. HM bioavailability was predicted in two stages (Scheme 1) as follows: (a) (i) First, the immobilization efficiency of soil and crops was predicted using an RF model, and (b) HM concentrations were calculated based on predicted immobilization efficiency to estimate crop uptake (CCU) and bioaccumulation factors (BAFs) after biochar immobilization. As shown in Figure 4, the accuracy of predicting CCU and BAFs by the developed RF model can be illustrated from the prediction set. It was determined that the R^2^ value for the RF model for CCU was 0.7338, which was higher than the R^2^ value for the BAF model (0.6997). The RF model was found to be effective in predicting the CCU and BAFs in the soil–crop systems containing biochar amendment. It has been established that RF exhibits the most reliable performance for predicting BAFs in soil–crop systems for all HMs, such as Cd, Pb, Cu, and Zn, with R^2^ values ranging from 0.17 to 0.84 [66]. Moreover, Zhu et al. [14] found that the RF model showed extremely high accuracy in predicting adsorption efficiency (R^2^ = 0.973). Comparing HM bioavailability in soil–crop systems with HM adsorption efficiency in water, there was a lack of accuracy in predicting HM bioavailability. Several factors affected the effectiveness of biochar as a soil amendment in soil–crop systems. For instance, the mixing depth of biochar had a significant influence on the uptake of HMs by crops; increasing the amount of fertilizer used (N, P, K dosages) adversely affected biochar’s effectiveness in soil–crop systems. Limited by the economic and labor costs, some factors such as biochar physical and chemical properties (pH and CEC), fertilization, and agricultural management policy were not recorded in most of the previous fields or pot experiments. 

The results showed that biochar effectively reduced the movement of HMs in the soil–crop systems. The content of HMs in the soil varies widely. Biochar used in soil elevates the pH and efficiently binds HMs. The research used four models to assess the efficiency of HM immobilization—LR, PLS, SVR, and RF. According to the data, the RF model had greater accuracy and relevance. The soil parameters have a considerable impact on immobilizing HMs with biochar. The study found that the efficacy of immobilization methods is dependent on the properties of biochar and the rate at which it is applied. In fact, the levels of soil organic carbon are directly proportional to the concentrations. Biochar reduces plant absorption of soil humus and HMs, which hinders pollutant assimilation. The RF model demonstrated superior performance compared to other models in predicting BAFs for all HMs in the soil–crop systems. Therefore, further research is needed to consider more related factors and improve model performance. Nonetheless, our study indicated that ML models can help us understand HM’s fate in soil–crop systems after biochar amendments and identify the key variables affecting immobilization efficiency.

## 4. Conclusions

This research employed four distinct models (linear regression, partial least squares, support vector regression, and random forest) to predict the absorption of HMs in biochar-amended soils by plants. The study utilized existing data from the previously published literature. The findings demonstrated that the RF model had more reliability in predicting performance in comparison to other models. The findings indicated that the RF approach can predict the crop absorption of HMs, providing an R^2^ value of 0.5924. The characteristic analysis revealed that the concentration of HM available was the most crucial factor impacting the variables. The established RF model achieved a prediction accuracy of 0.7338 for CCU and 0.6997 for BAFs, as measured by the R^2^ values. The primary factors that had an impact were determined to be soil properties (including the presence of HMs, the type of HMs, and the amount of clay) and the dose and properties of biochar (such as the temperature at which it was produced by pyrolysis). Although the accuracy of predicting the availability of HMs in soil–crop systems was lower compared to their efficiency in being absorbed by water, our study indicates that machine learning models could be valuable in comprehending the fate of HMs in soil–crop systems when biochar is used as an amendment. Additionally, these models can help identify the key variables that influence the effectiveness of biochar as a soil amendment. Establishing predictive models is crucial for accurately predicting the effects of biochar on the transformation of HMs in polluted soils, with the aim of ensuring the long-term stability of both humans and the environment. Hence, it is essential to do additional research in the future to optimize the information related to many factors.

## Figures and Tables

**Table 1:** Evaluation of the model performance on the basis of 5-fold cross-validation.

Parameters	LR	PLS	RF	SVR
R^2^	0.2785	0.3007	0.5924	0.2421
MAE	0.2605	0.2587	0.1556	0.2366
MSE	0.1054	0.1022	0.0596	0.0966
RMSE	0.3247	0.3197	0.2441	0.3109

## Data Availability

Data are contained within the article.

## Supplementary Materials

The following supporting information can be downloaded at: https://www.mdpi.com/article/10.3390/toxics12080575/s1, Figure S1: Deviation of the predicted and experimental change of crop uptake (CCU). Data points are sorted in ascending order by values at the x-axis; Table S1: Summary information for quantitative covariates; Table S2: Parts of tissues number: Table S3: Crop sample number for different plant.
