# Supplementary material for: Predictive Machine Learning Model to Assess the Adsorption Efficiency of Biochar-Heavy Metals for Effective Remediation of Soil–Plant Environment
Source: Toxics. 2024 Aug 7;12(8):575. doi: 10.3390/toxics12080575 (PMC11359540; doi:10.3390/toxics12080575)
Supplement: Supplementary file 1 [file toxics-12-00575-s001.zip › toxics-3114912-supplementary.pdf]

Predictive Machine Learning Model to Assess the Adsorption Efficiency of Biochar-Heavy Metals for Effective Remediation of Soil–Plant Environment

Xiang Li [1,*], Bing Chen [2], Weisheng Chen [1], Yilong Yin [1], Lianxi Huang [1], Lan Wei [1], Mahrous Awad [3] and Zhongzhen Liu [1,*]

1 Key Laboratory of Plant Nutrition and Fertilizer in South Region, Ministry of Agriculture, Guangdong Key Laboratory of Nutrient Cycling and Farmland Conservation, Institute of Agricultural Resources and Environment, Guangdong Academy of Agricultural Sciences, Guangzhou 510640, China; 13538631591@163.com (W.C.); yilong516@163.com (Y.Y.); hlx4@163.com (L.H.); 13450364816@163.com (L.W.)

2 Institute of Animal Science, Guangdong Academy of Agricultural Sciences, Collaborative Innovation Center of Aquatic Sciences, Key Laboratory of Animal Nutrition and Feed Science in South China, Ministry of Agriculture and Rural Affairs, Guangdong Provincial Key Laboratory of Animal Breeding and Nutrition, Guangzhou 510640, China; chenbing114@163.com

3 Department of Soils and Water, Faculty of Agriculture, Al-Azhar University, Assiut 71524, Egypt; mahrousawad.4419@azhar.edu.eg

* Correspondence: lixiang142213@163.com (X.L.); lzzgz2001@163.com (Z.L.)

Table S1. Summary information for quantitative covariates.

Variable	Min	Mean	Max	The first quartile (Q1)	Median	The third quartile (Q3)
Application rate of biochar (%)	0.22	3.14	10	1	3	5
Pyrolysis temperature (℃)	300	500	750	450	500	550
Biochar pH	8.2	10.1	10.53	9.4	10.0	10.4
Soil pH	4.71	6.28	8.06	5.7	6.01	6.8
SOC (g/kg)	0.045	16.54	190	6.3	12.27	15
Available Cd (mg/kg)	0.01	2.88	45	-	-	-
Available Pb (mg/kg)	0.024	4.81	15.45	-	-	-
Available Cu (mg/kg)	0.042	10.00	37.95	-	-	-
Available Zn (mg/kg)	0.15	84.41	295.63	-	-	-
Total Cd (mg/kg)	0.030	24.05	393.20	-	-	-
Total Pb (mg/kg)	4.50	996.26	4626	-	-	-
Total Cu (mg/kg)	3.20	347.83	693	-	-	-
Total Zn (mg/kg)	47.29	684.90	2226	-	-	-

Table S2. Parts of tissues number.

Parts of tissues	Sample Number
shoot	99
root	112

Table S3. Crop sample number for different plant.

Crops	Sample Number
Rice	8
Wheat	23
Corn	40
Vegetable	140

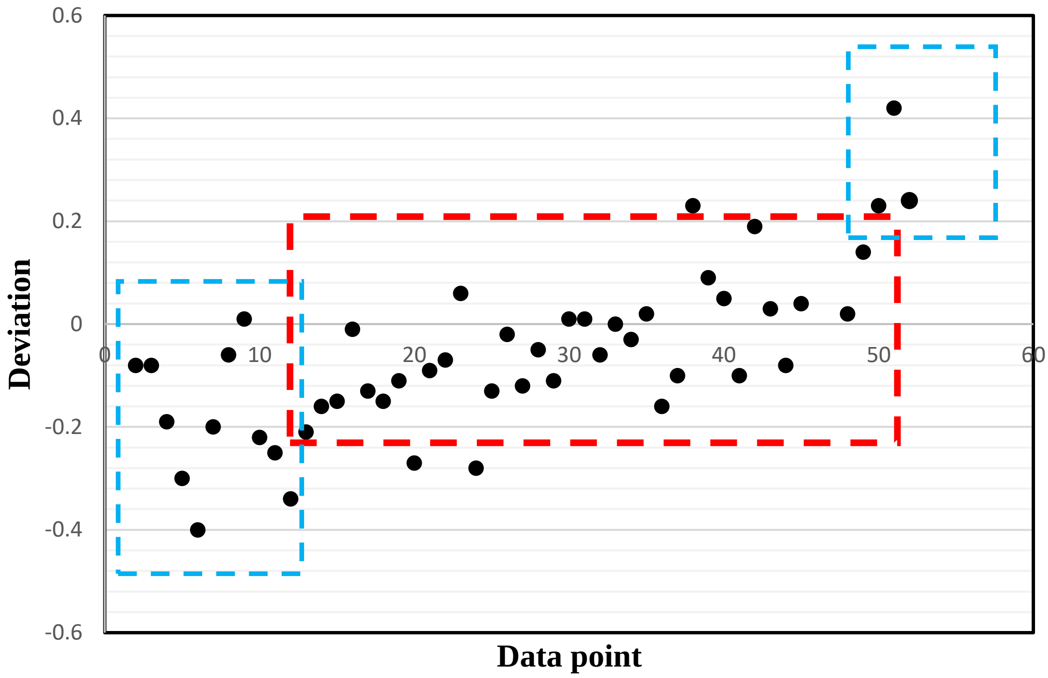

Figure S1. Deviation of the predicted and experimental change of crop uptake (CCU). Data points are sorted in ascending order by values at the x-axis.
